# Lower Oligomeric Form of Surfactant Protein D in Murine Acute Lung Injury Induces M1 Subtype Macrophages Through Calreticulin/p38 MAPK Signaling Pathway

**DOI:** 10.3389/fimmu.2021.687506

**Published:** 2021-08-16

**Authors:** Dandan Li, Linyue Pan, Xiaoju Zhang, Zhilong Jiang

**Affiliations:** ^1^Department of Pulmonary and Critical Care Medicine, Henan Provincial People’s Hospital, People’s Hospital of Zhengzhou University, Zhengzhou, China; ^2^Department of Pulmonary Medicine, Zhongshan Hospital, Fudan University, Shanghai, China

**Keywords:** acute lung injury, macrophages, surfactant protein D, calreticulin, signal regulatory protein-alpha

## Abstract

Surfactant protein D (SP-D) plays an important role in innate and adaptive immune responses. In this study, we found that the expression of total and de-oligomerized SP-D was significantly elevated in mice with lipopolysaccharide (LPS)-induced acute lung injury (ALI). To investigate the role of the lower oligomeric form of SP-D in the pathogenesis of ALI, we treated bone marrow-derived macrophages (BMDMs) with ALI-derived bronchoalveolar lavage (BAL) and found that SP-D in ALI BAL predominantly bound to calreticulin (CALR) on macrophages, subsequently increasing the phosphorylation of p38 mitogen-activated protein kinase (MAPK) and expression of interleukin (IL)-6, tumor necrosis factor (TNF)-alpha, IL-10, and CD80. However, anti-SP-D (aSP-D) and anti-calreticulin (aCALR) pretreatment reversed the SP-D binding and activation of macrophages induced by ALI BAL or de-oligomerized recombinant murine SP-D (rSP-D). Lack of signal transducer and activator of transcription (STAT)6 in STAT6-/- macrophages resulted in resistance to suppression by aCALR. Further studies in an ALI mouse model showed that blockade of pulmonary SP-D by intratracheal (i.t.), but not intraperitoneal (i.p.), administration of aSP-D attenuated the severity of ALI, accompanied by lower neutrophil infiltrates and expression of IL-1beta and IL-6. Furthermore, i.t. administration of de-oligomerized rSP-D exacerbated the severity of ALI in association with more pro-inflammatory CD45+Siglec-F(-) M1 subtype macrophages and production of IL-6, TNF-alpha, IL-1beta, and IL-18. The results indicated that SP-D in the lungs of murine ALI was de-oligomerized and participated in the pathogenesis of ALI by predominantly binding to CALR on macrophages and subsequently activating the pro-inflammatory downstream signaling pathway. Targeting de-oligomerized SP-D is a promising therapeutic strategy for the treatment of ALI and acute respiratory distress syndrome (ARDS).

## Introduction

Acute lung injury (ALI) and its more severe form, acute respiratory distress syndrome (ARDS), are defined as severe complications with systemic inflammatory responses in the air spaces and lung parenchyma. Surfactant protein D (SP-D) is a collagen-containing C-type lectin and synthesized primarily by alveolar type II cells and unciliated bronchial epithelial cells. However, serum SP-D is significantly increased in ALI/ARDS due to injury to the alveolar epithelial barrier and SP-D leakage into the blood circulation system. Thus, SP-D is proposed as a potential biomarker for the diagnosis and evaluation of ALI/ARDS (1–3). SP-D has diverse roles in different animal models under physiological and pathological conditions (4–6). In addition to its role in promoting macrophage phagocytosis and clearing invading pathogens and dead cells (7, 8), SP-D is also able to suppress macrophage activation through SIRP-alpha (9, 10). Thus, lack of SP-D in SP-D-/- mice induced lung fibrosis, alveolar epithelial injury, and early development of emphysema (11–13). Administration of surfactant proteins or recombinant fragments of SP-D can attenuate the severity of lung injury and asthma in mice (14–16).

In addition to the immunosuppressive function of SP-D, a recent *in vivo* study also indicated the pro-inflammatory function of SP-D. For example, lack of SP-D induced smaller atherosclerotic plaques, which might be caused by a decreased systemic inflammation (17). The dual roles of SP-D in different animal models and pathological conditions may be related to the distinct protein structure under different microenvironments. Multimeric SP-D is a dodecameric isoform, with N-termini of SP-D hidden in the center (triple-helical collagen region) and C-termini on the surface [carbohydrate recognition domain (CRD)]. The hydrophobic N-termini of SP-D are responsible for multimer SP-D assembly and opsonic activity by stabilizing disulfide bonds (18). It has been documented that dodecameric SP-D exerts anti-inflammatory properties by binding to resident macrophages with globular heads in a calcium- and carbohydrate-dependent manner (19, 20), participating in pathogen clearance through the formation of neutrophil extracellular trap (NET)-mediated bacterial trapping (21). However, oxidative stress under pathological and pro-inflammatory conditions can disassemble the dodecameric SP-D protein and form de-oligomerized SP-D. Recent studies have shown that de-oligomerized SP-D was significantly increased in an asthmatic mouse model (22, 23). Under oxidative stress conditions, cysteine residues in the hydrophobic tail domain of SP-D are S-nitrosylated, resulting in multimeric SP-D dissociation into trimeric or monomeric forms (6, 24). The head of the disassembled SP-D is incapable of eliciting anti-inflammatory function after occupation by pathogens, whereas the tail of the disassembled SP-D is released from the multimeric SP-D structure and possibly interacts with the calreticulin (CALR)/CD91 receptor complex on macrophages, subsequently activating macrophages (4, 6). Thus, it is important to maintain an optimal balance between intact and disassembled SP-D in lung tissues under pathological conditions.

Although SP-D expression is significantly elevated in ALI/ARDS, it remains unclear whether elevated endogenous SP-D participates in the development of ALI. To address this issue, we treated macrophages with SP-D derived from ALI BAL and de-oligomerized SP-D. The results revealed that de-oligomerized SP-D activated macrophages and promoted polarization of CD45+Siglec-F(-) M1 subtype macrophages through CALR signaling. Administration of anti-SP-D (aSP-D) antibody effectively attenuated the development of ALI and suppressed the activation of macrophages. Therefore, targeting endogenous SP-D may be a promising therapeutic strategy in the treatment of ALI/ARDS.

## Materials and Methods

### Cell Cultures

Bone marrow cells were flushed from the femurs and tibiae of mice and cultured in RPMI1640 culture medium supplemented with 10% fetal bovine serum (FBS) and 20% conditional media of NIH3T3 cells for 6 days to obtain bone marrow-derived macrophages (BMDMs). The murine macrophage cell line RAW264.7 cells were cultured in Dulbecco’s modified Eagle’s medium (DMEM) supplemented with 10% FBS.

### Mice and Treatment

Here, 10−12-week-old C57BL/6 male mice with ALI were established by intratracheal (i.t.) injection of 5 mg/kg lipopolysaccharide (LPS, *Escherichia coli* O55:B5; Sigma, St. Louis, MO, USA) in conjunction with or without 0.4 mg mouse aSP-D antibody/kg (sc-25324; Santa Cruz Biotech, CA, USA) or 0.3 mg/kg recombinant murine SP-D with endotoxin contamination less than 0.1 ng/μg (1 IEU/μg) (rSP-D, 6×His, AP75514; Signalway Antibody, College Park, MD, USA). The mice were treated with phosphate buffered saline (PBS) and immunoglobulin G (IgG)/LPS as controls. Two days after treatment, BAL and lung tissues were collected for analysis. All animals were housed and treated according to the guidelines of the Institutional Animal Care and Use Committee of Fudan University, Zhongshan Hospital, China. All experiments were approved by the committee.

### Flow Cytometry Assay

The 0.3 × 10^6^ cell suspensions of lung digests or BAL were incubated with an antibody cocktail containing PE-anti-CD45, PerC-Cy5-anti-F4/80, PE-Cy7-anti-Ly6G, APC-Cy7-anti-CD11b, and BV421-anti-Siglec-F (BD Biosciences, Franklin Lakes, NJ, USA; eBiosciences, San Diego, CA, USA). After incubation with the antibody cocktail for 40 min at room temperature, the cells were washed twice with PBS. The stained cells were analyzed by a BD FACSAria™ III instrument and BD FACSDiva™ software (BD Biosciences, San Jose, CA, USA). All data were analyzed using FlowJo software, version 8.8.4 (Tree Star Inc., Ashland, OR, USA).

### Cell Immunostaining Assay

RAW264.7 cells or BMDMs were pretreated with or without 2 µg/ml rabbit anti-calreticulin (aCALR; Abcam, Cambridge, MA, USA), anti--signal regulatory protein (SIRP)-alpha (aSIRP) antibody (Burlingame, CA, USA), 20 µM glibenclamide [GLMD, Nucleotide-binding oligomerization domain, leucine rich repeat and pyrin domain containing proteins (NLRP)3 inflammasome inhibitor], 20 µM pyrrolidine dithiocarbamate [PDTC, nuclear factor (NF)-κB inhibitor; Sigma- Aldrich, St. Louis, MO, USA) and 20 µM SB203580 [SB, p38 mitogen-activated protein kinase (MAPK) inhibitor; Cell Signaling Technology, Danvers, MA] for 1 h. Goat IgG isotype (R&D Systems Inc., Minneapolis, MN, USA) was used as a control. The pretreated cells were then incubated with 2 µg/ml rSP-D or BAL from naive or ALI mice containing endogenous SP-D (Naive BAL or ALI BAL, 1:3 dilution) for 24 h. The treated cells were used for immunostaining assay. Briefly, the cells were fixed with 4% paraformaldehyde for 10 min, permeabilized with 0.5% Triton X-100, and blocked with 5% goat serum for 30 min. Primary rabbit anti-mouse NLRP3, p38 MAPK, SIRP-alpha, and CALR were added to the cells overnight at 4°C. After washing with PBS, the cells were incubated with Cy3-conjugated anti-rabbit IgG antibody at room temperature for 1 h. After the nuclei were stained with 4′,6-diamidino-2-phenylindole (DAPI) for 3 min, the cells were visualized for positive staining under fluorescence microscope (Eclipse E800; Nikon, Melville, NY, USA).

### ELISA

The concentrations of tumor necrosis factor (TNF)-alpha, interleukin (IL)-1beta, IL-18, IL-6, and IL-10 were measured by ELISA kit (R&D Systems), according to the manufacturer’s instructions. SP-D was measured by ELISA kit (Signalway Antibody, College Park, MD, USA), and data were presented as SP-D ng/mg lung protein.

### Surfactant Protein D Cell Binding Assay

Here, 2 µg/ml rSP-D (6×His) was mixed with or without 2 µg/ml aSP-D in RPMI 1640 for 30 min at 37°C. Macrophages were treated with 2 µg/ml rSP-D or mixture of rSP-D/aSP-D for 24 h. The treated cells were fixed with 4% paraformaldehyde for 10 min and incubated with Cy3-conjugated anti-6×His tag antibody (Rockland Immunochemicals, Limerick, PA) for 30 min. The stained cells were visualized for SP-D binding under fluorescence microscope.

### Native Gel Electrophoresis for Surfactant Protein D

Here, 5 μg cell-free BAL protein per lane was mixed with 1× loading buffer without sodium dodecyl sulfate (SDS) and then resolved in 10% acrylamide/Bis gel without SDS, according to previous reports (22, 23). Glyceraldehyde 3-phosphate dehydrogenase (GAPDH) in cell-free BAL samples was not used as a loading control in Western blot analysis because GAPDH content in BAL was changed in response to lung inflammation (25, 26). After native gel electrophoresis, the proteins in the gels were transferred to nitrocellulose membranes. The blots were incubated with mouse anti-human SP-D (1:500 dilution) for 2 h, followed by incubation with horseradish peroxidase (HRP)-conjugated anti-mouse IgG for 1 h. After washing with TBST buffer, the blots were developed by enhanced chemiluminescence (ECL) substrate solution (Amersham Biosciences, Piscataway, NJ, USA).

### Statistical Analysis

Results are presented as dot plots or mean ± standard error (SE) of each group. All data were analyzed by one-way ANOVA followed by Tukey’s multiple comparisons or two-tailed unpaired Student’s *t-*test between two groups. Differences were considered statistically significant at p < 0.05.

## Results

### Surfactant Protein D Was Increased in Mice With Acute Lung Injury

**Figure 1:**
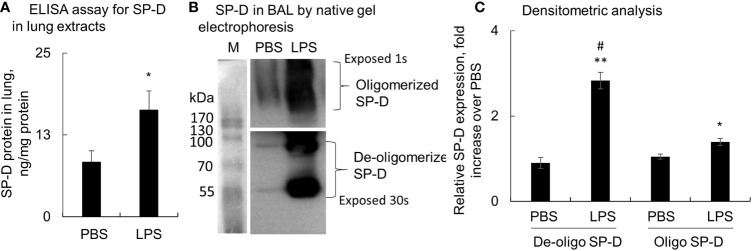
Lipopolysaccharide (LPS) induced more de-oligomerization of surfactant protein D (SP-D) in mice with acute lung injury (ALI). Mice with ALI were established by intratracheal (i.t.) administration of 5 mg/kg LPS for 2 days. **(A)** SP-D content in lung protein extracts was measured by ELISA. Value was normalized to the total protein amount in lung extracts and presented as mean ± SE, *p < 0.05 *vs*. those in mice treated with PBS control, n = 5. **(B)** SP-D structure in bronchoalveolar lavage (BAL) of phosphate buffered saline (PBS) and LPS-treated mice was analyzed by native gel electrophoresis. Multimeric or oligomerized SP-D was on the top of the gel. The de-oligomerized SP-D was resolved as 86 and 43 kDa. **(C)** Quantitative analysis of de-oligomerized and oligomerized SP-D by measuring densitometric value on ImageJ software. Data was presented as fold increase of each form of SP-D densitometric value in LPS group over those in the PBS control group, mean ± SE, *p < 0.05, **p < 0.01 *vs*. PBS control, ^#^p < 0.05 *vs*. oligomerized SP-D in LPS group, n = 5.

Lung tissues and BAL were collected from mice with LPS-induced ALI, and SP-D levels were evaluated by ELISA and Western blot analysis. The results showed that total SP-D was significantly elevated in the lung protein extracts of ALI mice compared to the PBS-treated control mice (p < 0.05, n = 5) (**Figure 1A**). In addition, we observed around 3-fold increased low molecular weight of SP-D (de-oligomerized SP-D) with size approximately 43−86 kDa in BAL of ALI mice compared to that in control mice. The high molecular weight of SP-D around 516 kDa (oligomerized SP-D) was also elevated around 1.4-fold in BAL of ALI mice compared to that in control mice (p < 0.05; **Figures 1B, C**). Thus, LPS significantly induced more de-oligomerized SP-D than oligomerized SP-D in mice with ALI relative to the mice treated with PBS (p < 0.05). The effects were possibly caused by de-oligomerization of dodecameric (516 kDa) to dimeric and monomeric SP-D (43 and 86 kDa) in mice with ALI.

### Anti-Surfactant Protein D Reduced Acute Lung Injury Mouse-Derived Surfactant Protein D Binding and Macrophage Activation

**Figure 2:**
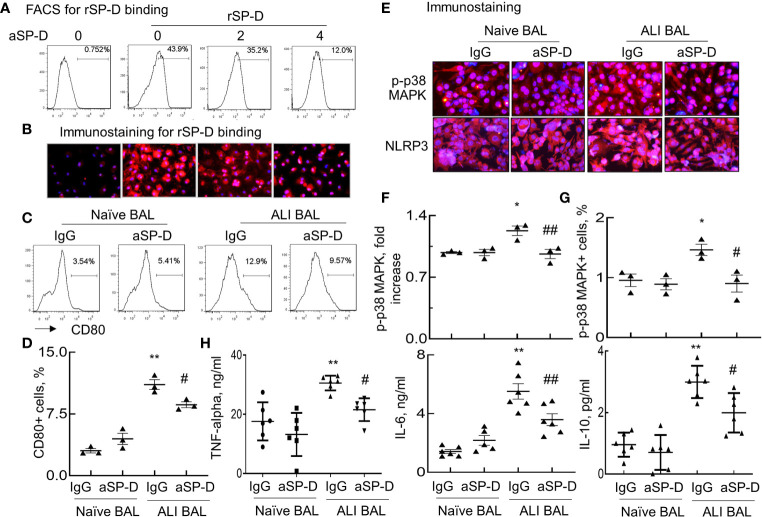
Anti-surfactant protein D (aSP-D) reduced acute lung injury (ALI) mice-derived SP-D binding and activation of macrophages. RAW264.7 cells were treated with 2 µg/ml recombinant murine SP-D (rSP-D) (6×His) pre-neutralized with 2, 4 µg/ml aSP-D. The cells that were untreated (0 group) or treated with 2 µg/m rSP-D (6×His) preincubated with 2 µg/ml immunoglobulin G (IgG) (0/rSP-D group) were used as controls. **(A)** Flow cytometry analysis for rSP-D binding on treated RAW264.7 cells by Cy3-conjugated anti-His antibody. **(B)** Immunostaining for rSP-D binding to the treated cells (red). Representative photograph with magnification ×200. **(C)** Flow cytometry analysis for macrophage activation induced by SP-D of bronchoalveolar lavage (BAL). RAW264.7 cells were treated with BAL of naive and ALI mice containing endogenous SP-D (naive BAL or ALI BAL) for 24 h. SP-D in BAL was pre-neutralized with 4 µg/ml aSP-D or IgG at 37°C for 1 h. Representative histogram for CD80 expression in the treated cells. **(D)** Quantitative analysis for CD80 expression in the treated cells. **(E)** Immunostaining for p-p38 mitogen-activated protein kinase (MAPK) and Nucleotide-binding oligomerization domain, Leucine rich Repeat and Pyrin domain containing Proteins (NLRP)3 expression in the treated cells. Red presents positively stained cells. Nuclei are stained with blue 4′,6-diamidino-2-phenylindole (DAPI). Representative photograph with magnification ×200. **(F)** Quantitative analysis of p-p38 MAPK fluorescence intensity in the stained cells by ImageJ software. Data were presented as mean increases of fluorescence intensity over IgG in naive BAL control. **(G)** Flow cytometry analysis of p-p38 MAPK in the treated cells. Data were presented as mean percentage of positively stained cells. **(H)** ELISA analysis for the expression of tumor necrosis factor (TNF)-alpha, interleukin (IL)-6, and IL-10 in the supernatants of the treated cells. *p < 0.05, **p < 0.01 *vs*. IgG/naive BAL group; ^#^p < 0.05, ^##^p < 0.01 *vs*. IgG/ALI BAL group for all quantitative analyses.

To further confirm whether ALI mouse-derived SP-D bound and subsequently activated macrophages, RAW264.7 cells were respectively incubated with rSP-D or endogenous SP-D derived from BAL of naive and ALI mice (Naive BAL and ALI BAL) for 24 h. rSP-D was pre-neutralized with different concentrations of aSP-D at 37°C for 1 h. SP-D binding on the cell surface was detected using Cy3-conjugated anti-His antibody. Flow cytometry and immunostaining showed that rSP-D can effectively bind to the cells. However, pre-neutralization with aSP-D effectively inhibited rSP-D binding in a concentration-dependent manner (**Figures 2A, B**). Furthermore, there were increased CD80+ cells treated with ALI BAL that was significantly reduced by aSP-D pretreatment (p < 0.05). However, the suppressive effects were not observed in the cells treated with naive BAL (**Figures 2C, D**). Further analysis by immunostaining (**Figures 2E, F**) and flow cytometry (**Figure 2G**) also showed that ALI BAL significantly enhanced the expression of p-p38 MAPK and NLRP3 in the treated macrophages, which was significantly reversed by aSP-D pretreatment (p < 0.05). With the activation of macrophages, the production of TNF-alpha, IL-6, and IL-10 was elevated in the supernatants of macrophages following stimulation with ALI BAL, which was effectively reversed by aSP-D pretreatment (**Figure 2H**). Therefore, aSP-D pretreatment suppressed the pro-inflammatory cytokine expression in ALI BAL-treated macrophages, indicating pro-inflammatory function of endogenous SP-D in ALI BAL.

### Blocking Calreticulin Reduced the Binding and Activation of Macrophages by Surfactant Protein D in Acute Lung Injury Mice

**Figure 3:**
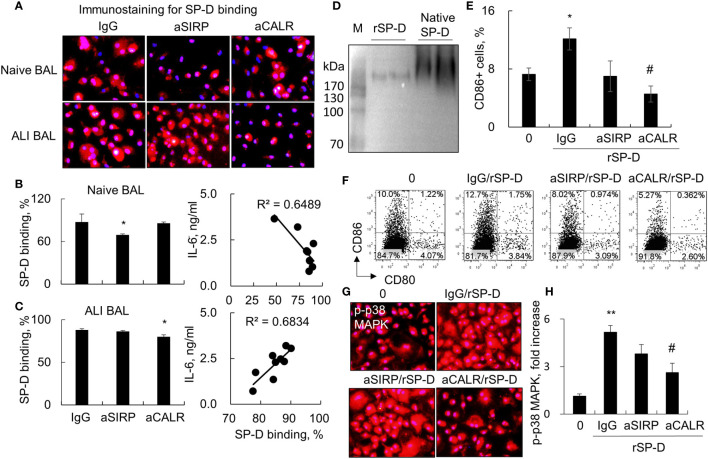
Blocking calreticulin activity predominantly reduced binding and activation of macrophages by acute lung injury (ALI) mouse-derived surfactant protein D (SP-D). **(A)** Immunostaining analysis for bronchoalveolar lavage (BAL)-derived endogenous SP-D binding on macrophages pretreated with anti-SIRP (aSIRP)-alpha and anti-calreticulin (aCALR). RAW264.7 cells were treated with naive and ALI mouse-derived BAL (1:3 dilution) for 24 h. The SIRP-alpha and calreticulin activities were pre-blocked with 2 µg/ml aSIRP or aCALR (Abcam, CA, USA) for 1 h. SP-D binding was detected by incubation with mouse anti-SP-D antibody (dilution 1:250) and followed by Cy3-conjugated anti-mouse immunoglobulin G (IgG) (dilution 1:1,000). Red indicates positive staining. Representative photograph with magnification ×200. **(B, C)** Quantitative analysis of SP-D binding of naive and ALI BAL by flow cytometry analysis and correlation with interleukin (IL)-6 expression. Data are presented as mean ± SE. n = 3, *p < 0.05 vs. IgG control. **(D)** Western blot analysis for recombinant murine SP-D (rSP-D) protein structure by native gel electrophoresis. Native SP-D is derived from BAL of naive mice. **(E)** Quantitative analysis for the expression of CD86 in treated RAW264.7 cells. *p < 0.05 vs. 0 group; ^#^p < 0.05, ^##^p < 0.01 vs. IgG/rSP-D group. **(F)** Representative histogram for the expression of CD80 in the treated cells. **(G, H)** Immunostaining and quantitative analysis for the expression of p-p38 mitogen-activated protein kinase (MAPK) in treated cells. Representative photograph with magnification ×200. Quantitative analysis of p-p38 MAPK positively stained cells by ImageJ software, and data are presented as mean increase of fluorescence intensity over control ± SE, n = 3, **p < 0.01 *vs*. 0 group; ^#^p < 0.05 *vs*. IgG/rSP-D group.

SP-D modulates diverse biological functions possibly *via* SIRP-alpha and CALR signaling (4). To determine whether SIRP-alpha and CALR signaling are involved in the downstream signaling of SP-D in ALI BAL, we pre-blocked SIRP-alpha and CALR activities on macrophages with 2 µg/ml aSIRP or aCALR (Abcam, CA, USA) for 1 h. Then, the pretreated cells were treated with SP-D of naive or ALI BAL for 24 h. The results of immunostaining and flow cytometry analysis showed that aSIRP pretreatment effectively suppressed the SP-D binding activity of naive BAL but not of ALI BAL. Due to the increased SP-D in ALI BAL, we observed more SP-D binding activity from ALI BAL than SP-D binding from naive BAL. However, the increased binding activity was reduced by aCALR, but not by aSIRP pretreatment. The results revealed that SP-D from ALI BAL competed with aCALR for CALR binding on macrophages, whereas SP-D from naive BAL competed with aSIRP for SIRP-alpha binding (**Figures 3A–C**). Therefore, SP-D from ALI BAL-activated macrophages predominantly binds to CALR, whereas SP-D from naive BAL predominantly binds to SIRP-alpha on macrophages. Consistently, we observed that SP-D of ALI BAL increased the IL-6 expression in macrophages, and SP-D binding activity was positively correlated with IL-6 expression level. In contrast, SP-D from naive BAL reduced IL-6 expression in macrophages, and SP-D binding activity was negatively correlated to IL-6 expression level (**Figures 3B, C**; p < 0.05, right panel). The results indicated the opposite function of SP-D from naive and ALI BAL on macrophages.

To further confirm that de-oligomerized SP-D in ALI BAL contributed to the pro-inflammatory function in macrophages, we treated macrophages with rSP-D, which is fused with 6×His tag at C-termini. Western blot analysis confirmed that rSP-D was approximately 258 kDa size of two homotrimer SP-D, smaller than 516 kDa size of dodecameric SP-D from BAL of naive mice (**Figure 3D**). The results indicated that the commercial rSP-D is de-oligomerized or not well assembled. The results of flow cytometry and immunostaining analysis revealed that rSP-D effectively increased the percentage of CD86+ cells and phosphorylation of p38 MAPK. Pretreatment of cells with aCALR significantly suppressed the percentage of CD86+ cells and activation of p38 MAPK in rSP-D-treated macrophages. However, the suppressive effects were not well pronounced in aSIRP-pretreated cells (**Figures 3E–H**). Additional study showed that the effects were not caused by cytotoxicity of aCALR or aSIRP because aCALR or aSIRP treatment did not impact cell viability (data not shown).

### Anti-Surfactant Protein D Suppressed Acute Lung Injury Bronchoalveolar Lavage-Induced Activation of Macrophages Through Calreticulin/Signal Transducer and Activator of Transcription 6 Signaling

**Figure 4:**
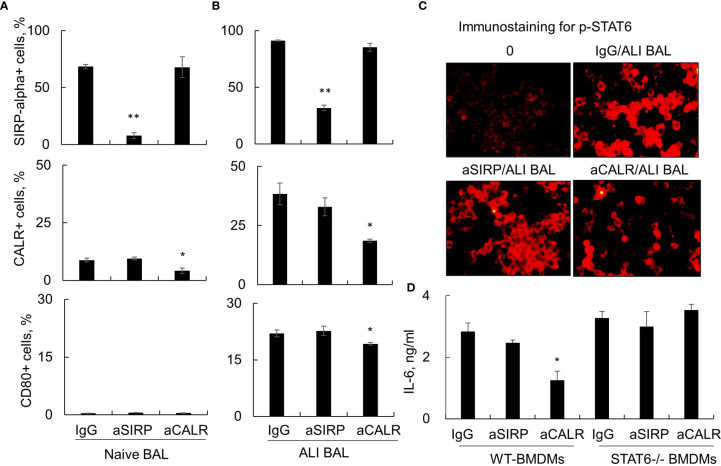
Anti-calreticulin (aCALR) did not suppress acute lung injury (ALI) bronchoalveolar lavage (BAL)-induced macrophage activation and interleukin (IL)-6 expression in signal transducer and activator of transcription (STAT)6-/- bone marrow-derived macrophages (BMDMs). **(A, B)** Flow cytometry analysis for the expression of SIRP-alpha, CALR, and CD80 on RAW264.7 cells. The cells were pretreated with 2 µg/ml aSIRP, aCALR, or immunoglobulin G (IgG) isotype control for 1 h prior to stimulation with naive or ALI BAL (1:3 dilution). Data were presented as mean ± SE, n = 3, *p < 0.05, **p < 0.01 *vs*. IgG control. **(C)** Immunostaining for activated STAT6 (p-STAT6 at Tyr641 residue) in the treated RAW264.7 cells. The cells were incubated with rabbit anti-p-STAT6 antibody (1:200 dilution) and followed by Cy3 conjugated anti-rabbit IgG (1:400 dilution). Representative photograph with magnification ×200. **(D)** ELISA analysis for the expression of IL-6 in the supernatants of treated wild-type (WT) and STAT6-/- derived BMDMs. Data were presented as mean ± SE, n = 3, *p < 0.05, **p < 0.01 *vs*. IgG control.

The Janus kinase (JAK)/signal transducer and activator of transcription (STAT)6 signaling pathway has been shown to play a regulatory role in immune responses (27, 28). To further determine whether STAT6 signaling was involved in the activation of macrophages by ALI BAL-derived SP-D, we stimulated macrophages from wild-type (WT) and STAT6-/- mice with naive or ALI BAL, respectively, pretreated with aSIRP and aCALR. Flow cytometry analysis showed that aSIRP and aCALR specifically blocked SIRP-alpha and CALR on macrophages. In addition, ALI BAL markedly increased the expression of CALR and CD80 but mildly increased the expression of SIRP-alpha. However, aCALR significantly attenuated the expression of CALR and CD80 induced by ALI BAL, indicating that some mediators including de-oligomerized SP-D in ALI BAL activated macrophages through CALR signaling (**Figures 4A, B**). Consistently, immunostaining and ELISA analysis revealed the activated STAT6, as well as enhanced expression of IL-6 by ALI BAL, that was effectively reversed by aCALR pretreatment (**Figures 4C, D**). However, aCALR pretreatment did not suppress the expression of IL-6 induced by ALI BAL in STAT6-/- BMDMs, even slightly increased their expression (**Figure 4D**). These results indicated that de-oligomerized SP-D in ALI BAL may activate macrophages through CALR/STAT6 signaling, and STAT6 is required for aCALR suppressive effects on macrophages.

### Recombinant Murine Surfactant Protein D Activated Macrophages Through p38 MAPK/NLRP3/NF-κB Signaling

To further investigate whether de-oligomerized SP-D activated macrophages through p38 MAPK, NLRP3, and NF-κB signaling, we pretreated macrophages with NLRP3, NF-κB, and p38 MAPK inhibitors, including GLMD, PDTC, and SB, respectively. The cells were then stimulated with rSP-D for 24 h. Flow cytometry analysis showed that rSP-D moderately increased the expression of CD80 and CD86. However, the effects were moderately attenuated by pretreatment with GLMD, PDTC, and SB (**Supplemental Figures S1A, B**). Similar results were also observed for the expression of TNF-alpha and IL-6 (**Supplemental Figures S1C, D**). The results indicated that p38 MAPK/NLRP3/NF-κB signaling was involved in the activation of macrophages and cytokine expression induced by rSP-D.

### Recombinant Murine Surfactant Protein D Enhanced the Severity of Murine Acute Lung Injury

**Figure 5:**
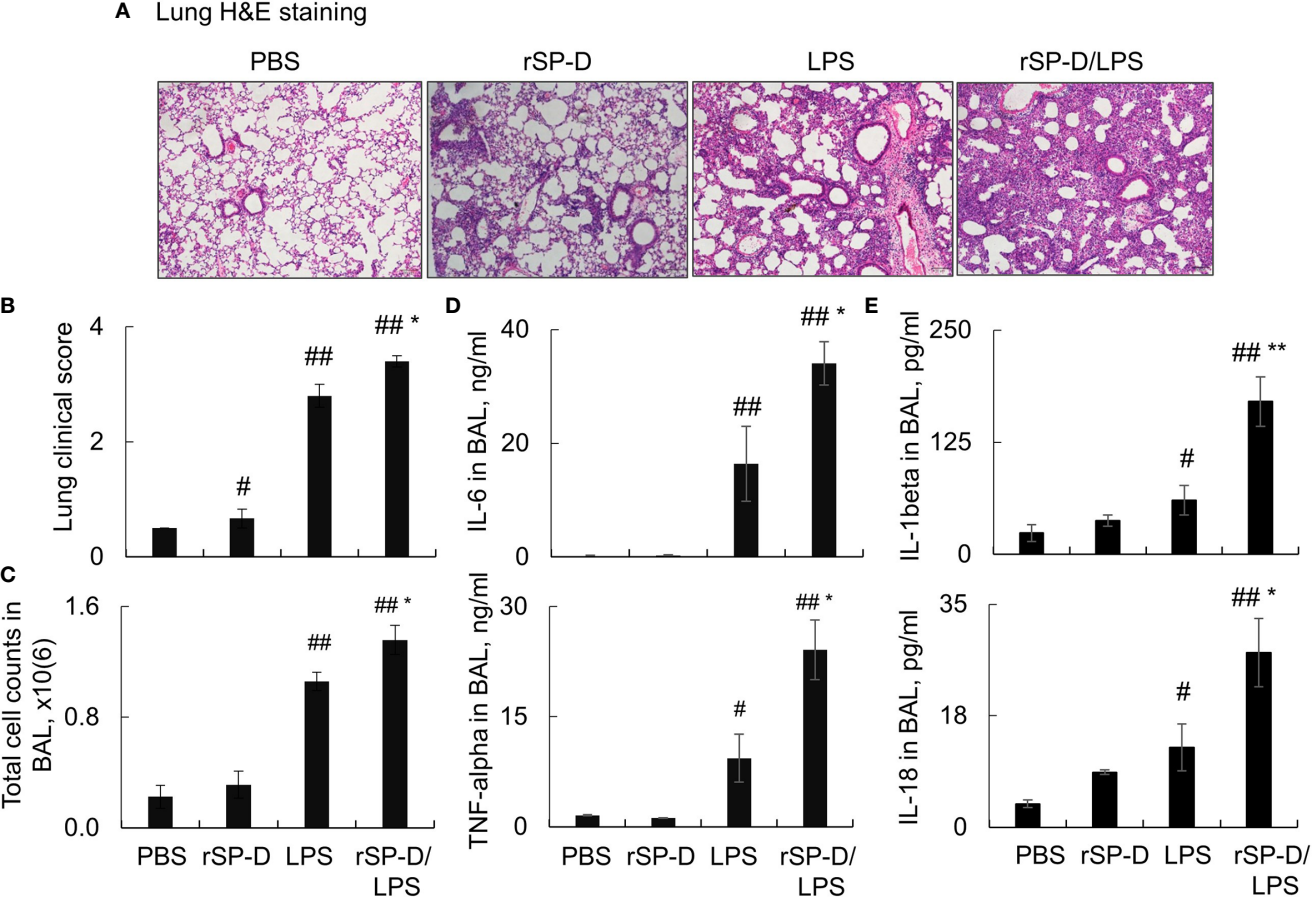
Recombinant murine surfactant protein D (rSP-D) enhanced the severity of acute lung injury (ALI) in mice. C57BL/6 mice were co-i.t. treated with both 0.3 mg/kg rSP-D and 5 mg/kg lipopolysaccharide (LPS) (rSP-D/LPS group) for 2 days. The mice treated with phosphate buffered saline (PBS), rSP-D, LPS alone were controls. **(A)** Lung histology by H&E staining. Representative photograph with magnification ×100. **(B)** Quantitative analysis of lung histology in each group. **(C)** Total cell counts in bronchoalveolar lavage (BAL). **(D, E)** ELISA assay for the expression of interleukin (IL)-6, tumor necrosis factor (TNF)-alpha, IL-1beta, and IL-18 in BAL. All data were presented as mean ± SE. n = 7 (LPS and rSP-D/LPS groups) or 3 (PBS and rSP-D groups). *p < 0.05, **p < 0.01 *vs*. immunoglobulin G (IgG)/LPS group; ^#^p < 0.05, ^##^p < 0.01 *vs*. PBS group.

To investigate the role of de-oligomerized SP-D in the development of ALI in mice, we i.t. co- treated C57BL/6 mice with both 0.3 mg/kg rSP-D and 5 mg/kg LPS (rSP-D/LPS group) for 2 days. Mice treated with PBS, rSP-D alone, or LPS were used as controls. As a result, we observed a moderately increased pathological lung inflammation in the rSP-D group compared to that in the PBS-treated control group. The pathological lung inflammation was significantly increased in the rSP-D/LPS group compared to that in the LPS alone-treated group, indicating synergistic effects of rSP-D in promoting lung inflammation (**Figures 5A, B**). The enhanced lung inflammation was accompanied by higher total cell counts (**Figure 5C**) and more expression levels of IL-6, TNF-alpha, IL-1beta, and IL-18 in BAL of mice in the rSP-D/LPS group than in the LPS group. In addition, the expression of IL-1beta and IL-18 was moderately increased in the rSP-D group compared to the PBS group. The results indicated the pro-inflammatory role of rSP-D in murine ALI (**Figures 5D, E**).

**Figure 6:**
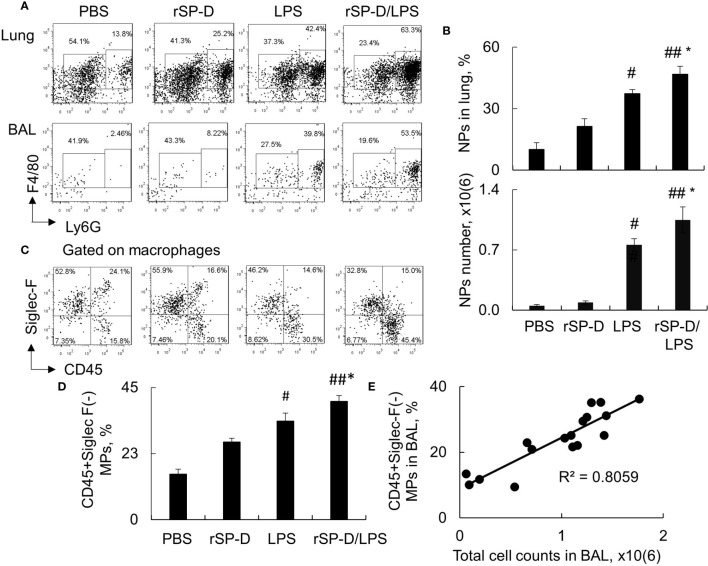
Recombinant murine surfactant protein D (rSP-D) increased neutrophil infiltration and CD45+Siglec-F(-) M1 subtype macrophages in the lung of mice with acute lung injury (ALI). **(A)** Flow cytometry analysis for Ly6G+ neutrophils (NPs) and Ly6G(-) macrophages (MPs) in the lung digests and bronchoalveolar lavage (BAL) of treated mice. Representative dot plot was shown. **(B)** Quantitative analysis of the percentage and absolute number of NPs in the lung tissues and BAL, respectively. * p < 0.05 *vs*. lipopolysaccharide (LPS) group; ^#^p < 0.05, ^##^p < 0.01 *vs*. phosphate buffered saline (PBS) group. **(C)** Flow cytometry analysis for CD45+Siglec-F(-) M1 and CD45+Siglec-F(+) M2 subtype MPs in BAL. Representative dot plot was gated on MP population. **(D)** Quantitative analysis of CD45+Siglec-F(-) subtype MPs in BAL. Data were presented as mean ± SE. *p < 0.05 *vs*. LPS group; ^#^p < 0.05, ^##^p < 0.01 *vs*. PBS group. **(E)** Correlation analysis between CD45+Siglec-F(-) M1 subtype MPs and total cell counts in BAL. Each dot presents the value of an individual mouse.

Consistent with the results above, our additional analysis by flow cytometry analysis revealed that rSP-D induced 2–3-fold more infiltration of F4/80+Ly6G+ neutrophils in the rSP-D/LPS group than that of the LPS group (**Figures 6A, B**). The percentage of neutrophils was moderately increased in rSP-D alone-treated mice associated with moderately increased percentage of CD45+Siglec-F(-) M1 subtype macrophages in the rSP-D group compared to that in the PBS group. The percentage of CD45+Siglec-F(-) M1 subtype macrophages was significantly higher in the rSP-D/LPS group than those in the LPS group (**Figures 6C, D**). Further analysis showed a positive correlation between CD45+Siglec-F(-) M1 subtype macrophages and total cell counts in BAL (**Figure 6E**). The results indicated that rSP-D promoted ALI and lung inflammation in association with CD45+Siglec-F(-) M1 subtype macrophage-biased polarization *in vivo*. CD45+Siglec-F(-) M1 subtype macrophages may contribute to the pathogenesis of ALI in mice.

### Intratracheal Administration of Anti-Surfactant Protein D Attenuated Acute Lung Injury and Lung Inflammation in Mice

**Figure 7:**
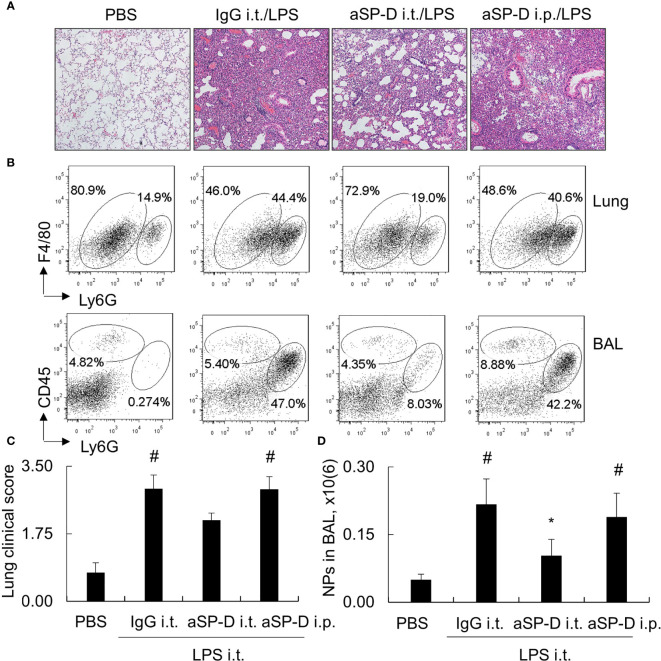
Intratracheal administration of anti-surfactant protein D (aSP-D) antibody attenuated the severity of acute lung injury (ALI) and inflammation in the lungs of mice. Here, 10−12-week-old C57BL/6 male mice were intratracheal (i.t.) or intraperitoneal (i.p.) co-treated with 0.4 mg aSP-D/kg and 5 mg/kg lipopolysaccharide (LPS) for 2 days (aSP-D i.t./LPS group and aSP-D i.p./LPS group). Mice treated with phosphate buffered saline (PBS) and immunoglobulin G (IgG)/LPS were controls. **(A)** Lung histology by H&E staining. Representative photograph with magnification ×100. **(B)** Flow cytometry analysis for neutrophils (NPs) and macrophages (MPs) in lung tissues and bronchoalveolar lavage (BAL). NPs were identified as Ly6G+ cells; MPs were identified as CD45+Ly6G(-) cells. **(C)** Quantitative analysis of lung pathological score. **(D)** Absolute number of NPs in BAL. *p < 0.05 *vs*. IgG/LPS group; ^#^p < 0.05 *vs*. PBS group.

**Figure 8:**
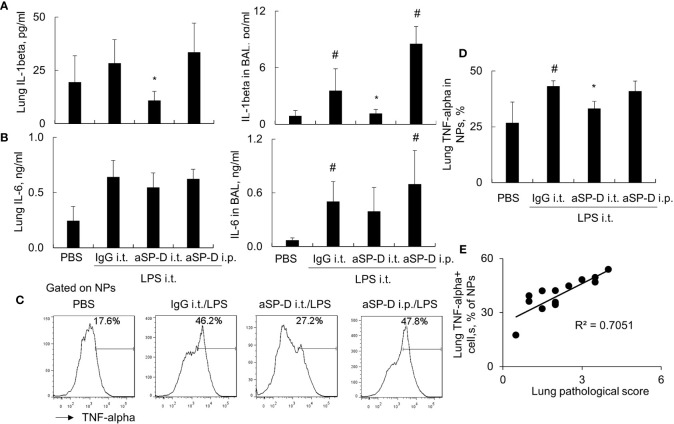
Intratracheal administration of anti-surfactant protein D (aSP-D) suppressed the expression of pro-inflammatory cytokines in mice with acute lung injury (ALI). **(A, B)** ELISA assay for the expression of interleukin (IL)-1beta and IL-6 in lung extracts and bronchoalveolar lavage (BAL) of each group. *p < 0.05 *vs*. immunoglobulin G (IgG)/lipopolysaccharide (LPS) group; ^#^p < 0.05 *vs*. phosphate buffered saline (PBS) group. **(C)** Intracellular staining for tumor necrosis factor (TNF)-alpha expression in neutrophils. TNF-alpha+ neutrophils (NPs) were gated on NPs. Representative histogram of each treatment was shown. **(D)** Quantitative analysis of TNF-alpha+ NPs. **(E)** Correlation analysis between TNF-alpha+ NPs and lung pathological score. Each dot presents the value of an individual mouse.

To investigate whether blockade of de-oligomerized SP-D in ALI attenuates ALI and lung inflammation *in vivo*, 10−12-week-old male C57BL/6 mice were treated with 0.4 mg i.t. or intraperitoneal (i.p.) aSP-D/kg mouse (sc-25324; Santa Cruz) in conjunction with 5 mg/kg LPS for 2 days (aSP-D i.t./LPS and aSP-D i.p./LPS groups). The mice treated with PBS (PBS group) or co-treated with both IgG and LPS were used as controls (IgG i.t./LPS group). The results of H&E staining and flow cytometry analysis revealed that i.t. aSP-D, but not i.p. aSP-D treatment, effectively reduced the severity of ALI, accompanied by attenuated infiltration of Ly6G+ neutrophils in lung tissues and BAL, compared to the mice in the IgG/LPS group (**Figures 7A–D**). Consistently, we observed moderately reduced expression of IL-1beta and IL-6 in the lung and BAL of the aSP-D i.t./LPS group, but not the aSP-D i.p./LPS group (**Figures 8A, B**). Further analysis by intracellular staining showed that neutrophils can express a high level of TNF-alpha in mice with ALI, which was significantly reversed by i.t. aSP-D, but not i.p. aSP-D co-treatment (**Figures 8C, D**). The expression of TNF-alpha in neutrophils was positively correlated with lung pathological score (**Figure 8E**), indicating the involvement of TNF-alpha expressing neutrophils in the development of ALI.

## Discussion

ARDS is a life-threatening ALI with a mortality rate of approximately 40% (29, 30). Macrophages and neutrophils are important cell components in lung tissues that critically participate in the development of ALI/ARDS (31, 32). Recent reports have shown that SP-D expression is elevated in lung tissues and the blood circulation system (2, 3, 33). The increase in pro-inflammatory cytokines and mediators is responsible for the upregulation of SP-D expression *in vivo* (22). Due to the anti-inflammatory role of multimeric SP-D, recent studies have shown that repetitive surfactant replacement therapy attenuated (14–16), but the lack of SP-D exacerbated, disease severity in some animal models (11, 12).

However, it was reported that multimeric SP-D can be de-oligomerized into trimeric or monomeric SP-D under oxidative stress (6, 23, 24). Different SP-D structures have distinct biological functions. Multimeric SP-D has phagocytotic and anti-inflammatory properties by interacting between SP-D global head (C-termini) and SIRP-alpha on macrophages or other resident cells. However, trimeric SP-D and monomeric SP-D have high pro-inflammatory activity by SP-D tail (N-termini) binding to CALR on macrophages or other resident cells (4, 20).

In ALI/ARDS, SP-D expression is increased due to oxidative stress. However, it is unclear whether the increased endogenous SP-D levels in ALI/ARDS are responsible for disease development. To address this issue, we in this study, for the first time, neutralized the endogenous SP-D in murine ALI by i.t. or i.p. injection of aSP-D neutralizing antibody and found that i.t. aSP-D, but not i.p. aSP-D, effectively reduced the severity of ALI and expression of pro-inflammatory cytokines, indicating the pro-inflammatory role of lung endogenous SP-D in ALI. The results confirmed the importance of pulmonary SP-D, but not circulating SP-D, in the development of ALI. Consistent with these results, the pro-inflammatory function of SP-D was also reported in other animal models, such as atherosclerosis (17) and acute kidney injury (AKI) (34), in which attenuated vessel plaques and AKI severity were observed in SP-D-/- mice. To further confirm the pro-inflammatory function of SP-D in ALI, we i.t. treated naive or ALI mice with low doses of de-oligomerized SP-D, rSP-D. As expected, we observed a moderately increased lung inflammation in naive mice and synergistically enhanced lung inflammation in the mice with ALI after i.t. treatment with rSP-D. However, the experiment was limited by low doses of rSP-D used in naive mice, and we expect that more lung inflammation would be achieved with higher doses of i.t. rSP-D administered in mice. Further *in vitro* studies have revealed the involvement of p38-MAPK, NLRP3, and NF-κB signaling in the activation and polarization of CD45+Siglec-F(-) M1 subtype macrophages because p38-MAPK, NLRP3, and NF-κB inhibitors reversed the activation and pro-inflammatory cytokine expression in the treated macrophages. Thereby, two homotrimer SP-D (rSP-D) activated macrophages predominantly through CALR/p38 MAPK signaling pathway, consistent with the previous report, in which trimeric SP-D failed to correct the pulmonary phospholipid accumulation and emphysema characteristic of SP-D knockout mice (35).

Our results are different from that of a previous report in which mice lacking SP-D developed more severe ALI (36). This discrepancy may be explained by lack of multimeric SP-D in the SP-D knockout mouse model because multimeric SP-D in lung tissues may be necessary to overcome excessive lung inflammation in mice with ALI. It would be essential to maintain an optimal balance between pulmonary multimeric and lower oligomeric form of SP-D in the pathogenesis of ALI. The underlying molecular mechanisms of aSP-D in attenuating ALI will be further investigated in the future.

To confirm the presence of de-oligomerized SP-D in the lungs of ALI mice, we analyzed the SP-D structure by native gel electrophoresis. As a result, we found that dodecameric/multimeric SP-D was at the top of the native gel, whereas de-oligomerized SP-D was resolved as smaller sizes of protein, predominantly 86 and 43 kDa. Both de-oligomerized SP-D and oligomerized SP-D were markedly increased in mice with ALI, with significantly more increases in de-oligomerized SP-D than oligomerized SP-D. A previous report by Yamazoe et al. (37) indicated that the correct oligomeric structure of SP-D is important in downregulating LPS-elicited inflammatory responses by suppressing LPS binding to its receptors on macrophages. We speculate that the lower oligomeric form of SP-D in ALI BAL did not effectively suppress LPS-induced activation of macrophages and the expression of pro-inflammatory cytokines due to its inefficient binding to LPS compared to multimeric SP-D in native BAL. In contrast, the de-oligomerized SP-D activated macrophages through binding to CALR on macrophages. Thus, endogenous SP-D in the ALI mouse model is predisposed to de-oligomerization by oxidative stress and is pathogenic.

Because it was previously assumed that SP-D can bind to both SIRP-alpha and CALR (4), we blocked SIRP-alpha and CALR activities on macrophages by pretreatment with antibodies against SIRP-alpha (aSIRP) or CALR (aCALR) to further determine whether ALI BAL-derived SP-D exerted pro-inflammatory function by binding to CALR or SIRP-alpha. The *in vitro* results revealed that pretreatment with aCALR, but not aSIRP, reduced ALI BAL-derived SP-D binding on macrophages, indicating the involvement of CALR in de-oligomerized SP-D-mediated pro-inflammatory function in ALI mice. In contrast, the binding activity of naive mouse-derived SP-D was reduced in the cells pretreated with aSIRP, but not aCALR. The results for the first time provided solid evidence that multimeric SP-D predominantly binds to SIRP-alpha and de- oligomerized SP-D including two homotrimer SP-D, dimeric SP-D and monomeric SP-D, that predominantly bind to CALR, supporting the previous assumption (4). Therefore, blocking CALR signaling would be a promising therapeutic strategy for ALI, as reported previously by our research group (32).

It is noted that blocking SIRP-alpha signaling by aSIRP pretreatment reduced the activation of macrophages to a certain degree, indicating a pro-inflammatory role of SIRP-alpha under a certain condition, though previous report showed the immune suppressive role of SIRP-alpha (4, 9). The pro-inflammatory effects of SIRP-alpha were further confirmed in an *in vivo* study, in which lack of SIRP-alpha in SIRP-alpha knockout mice significantly reduced the severity of ALI (unpublished data). Therefore, SIRP-alpha might have diverse functions in different animal models, as previously reported (34, 38, 39). SIRP-alpha signaling possibly participated in the pro-inflammatory function of endogenous SP-D in ALI mice.

To further define whether JAK/STAT6 signaling is involved in the SIRP-alpha or CALR-mediated downstream signaling pathway, we pretreated WT and STAT6-/- derived macrophages (BMDMs) with aSIRP and aCALR. The results showed that aCALR was more effective than aSIRP in suppressing the activation of WT BMDMs, but lack of STAT6 expression in STAT6-/- BMDMs resulted in resistance to the suppressive effects of aCALR, indicating the involvement of STAT6 signaling in aCALR-mediated suppression of macrophage activation. Our further study in the STAT6-/- mouse model revealed the immune regulatory function of STAT6 in mice with ALI because lack of STAT6 in STAT6-/- mice exacerbated ALI and induced more lung inflammation (unpublished data). Thus, de-oligomerized SP-D in ALI may activate STAT6 through CALR binding. The increased STAT6 activation suppressed macrophage activation and pro-inflammatory cytokine expression in feedback through suppressing p38 MAPK, NF-κB, and NLRP3 (**Supplementary Figure S2**).

Taken together, our study revealed that dodecameric SP-D was disassembled into de-oligomerized SP-D in ALI mice, subsequently inducing the polarization of CD45+Siglec-F(-) M1 subtype macrophages predominantly through CALR/p38 MAPK and downstream pro-inflammatory signaling. Targeting de-oligomerized SP-D and its downstream signaling pathways would be a promising approach in the treatment of ALI/ARDS.

## Data Availability Statement

The raw data supporting the conclusions of this article will be made available by the authors, without undue reservation.

## Ethics Statement

The animal study was reviewed and approved by the Institutional Animal Care and Use Committee of Fudan University, Zhongshan Hospital, China.

## Author Contributions

DL and LP participated in cell culture, ELISA, and Western blot analysis. XZ generated the hypothesis. ZJ is responsible for generating the hypothesis, performing flow cytometry, data analysis, animal experiments, manuscript writing, and revision and is responsible for all directions of the work. All authors contributed to the article and approved the submitted version.

## Funding

This work is funded by a research grant from the Natural Science Foundation of Shanghai to ZJ (19ZR1409000), the National Natural Science Foundation of China to XZ (81970077), and the Doctoral Scientific Research Foundation of Henan Provincial People’s Hospital, Zhengzhou, Henan, China, to DL (ZC20190166).

## Conflict of Interest

The funders had no role in the study design, data collection and analysis, decision to publish, or preparation of the manuscript. The authors declare that the research was conducted in the absence of any commercial or financial relationships that could be construed as a potential conflict of interest.

## Publisher’s Note

All claims expressed in this article are solely those of the authors and do not necessarily represent those of their affiliated organizations, or those of the publisher, the editors and the reviewers. Any product that may be evaluated in this article, or claim that may be made by its manufacturer, is not guaranteed or endorsed by the publisher.

## Supplementary Material

Supplementary Figure 1 Inhibition of p38 MAPK, NLRP3, and NF-κB signaling attenuated macrophage activation by rSP-D. **(A)** RAW264.7 cells were pretreated with 20 µM glibenclamide (GLMD), 20 µM PDTC, and 20 µM SB203580 (SB) for 1 h, followed by stimulation with 2 µg/ml rSP-D for 24 h. The expression of CD80 was analyzed by flow cytometry. Representative histograms of the treated cells are shown. **(B)** Quantitative analysis of CD80 and CD86 in treated cells. **(C, D)** ELISA analysis for TNF-alpha and IL-6 expression in the supernatants of treated cells. *p < 0.05 *vs*. 0 group; #p < 0.05, ##p < 0.01 *vs*. rSP-D group.

Supplementary Figure 2 Schematic diagram of SP-D signaling pathway in mice with ALI. SP-D is de-oligomerized in murine ALI or patients with ARDS due to oxidative stress. The de-oligomerized SP-D predominantly binds to Calreticulin (CALR), subsequently activating macrophages and expression of pro-inflammatory cytokines IL-6, TNF-alpha, IL-1beta and IL-18, through activation of p38 MAPK, NF-κB, NLRP3 and STAT6 signaling. STAT6 signaling suppresses activation of p38 MAPK, NF-κB and NLRP3 in feedback, prevents uncontrolled lung inflammation in mice with ALI.
