# Supplementary figures and images for: Lower Oligomeric Form of Surfactant Protein D in Murine Acute Lung Injury Induces M1 Subtype Macrophages Through Calreticulin/p38 MAPK Signaling Pathway
Source: Front Immunol. 2021 Aug 16;12:687506. doi: 10.3389/fimmu.2021.687506 (PMC8415422; doi:10.3389/fimmu.2021.687506)

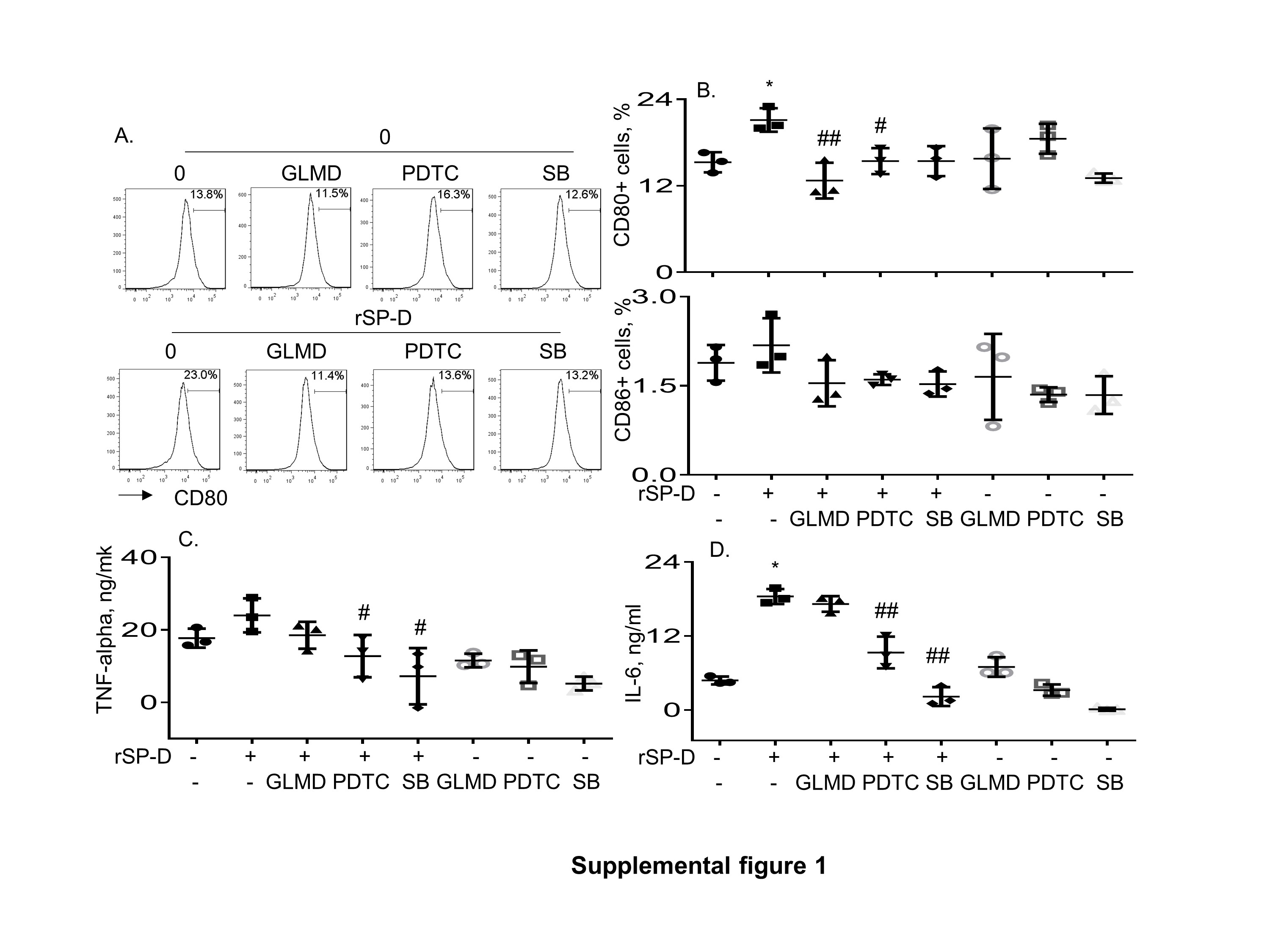

Supplement: Supplementary Figure 1 — Inhibition of p38 MAPK, NLRP3, and NF-κB signaling attenuated macrophage activation by rSP-D. (A) RAW264.7 cells were pretreated with 20 µM glibenclamide (GLMD), 20 µM PDTC, and 20 µM SB203580 (SB) for 1 h, followed by stimulation with 2 µg/ml rSP-D for 24 h. The expression of CD80 was analyzed by flow cytometry. Representative histograms of the treated cells are shown. (B) Quantitative analysis of CD80 and CD86 in treated cells. (C, D) ELISA analysis for TNF-alpha and IL-6 expression in the supernatants of treated cells. *p < 0.05 vs. 0 group; #p < 0.05, ##p < 0.01 vs. rSP-D group. [file Image_1.jpeg]

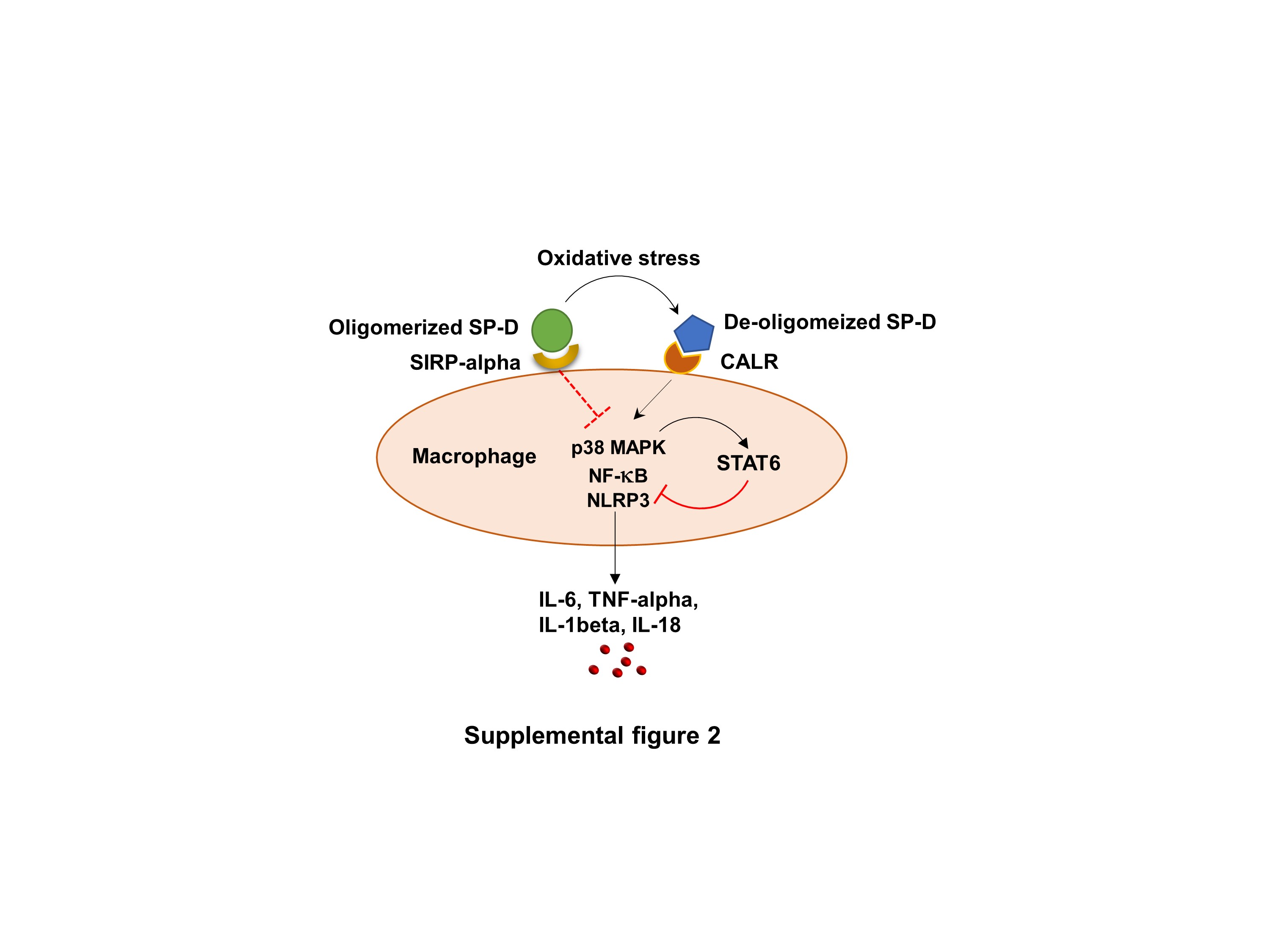

Supplement: Supplementary Figure 2 — Schematic diagram of SP-D signaling pathway in mice with ALI. SP-D is de-oligomerized in murine ALI or patients with ARDS due to oxidative stress. The de-oligomerized SP-D predominantly binds to Calreticulin (CALR), subsequently activating macrophages and expression of pro-inflammatory cytokines IL-6, TNF-alpha, IL-1beta and IL-18, through activation of p38 MAPK, NF-κB, NLRP3 and STAT6 signaling. STAT6 signaling suppresses activation of p38 MAPK, NF-κB and NLRP3 in feedback, prevents uncontrolled lung inflammation in mice with ALI. [file Image_2.jpeg]
